# HIV Prevalence and Factors Influencing the Uptake of Voluntary HIV Counseling and Testing among Older Clients of Female Sex Workers in Liuzhou and Fuyang Cities, China, 2016-2017: A Cross-Sectional Study

**DOI:** 10.1155/2020/9634328

**Published:** 2020-02-20

**Authors:** Qi Zhang, Yuan-Sheng Fu, Xue-Mei Liu, Zhi-Qiang Ding, Ming-Qiang Li, Yin-Guang Fan

**Affiliations:** ^1^Department of Epidemiology and Biostatistics, School of Public Health, Anhui Medical University, 81 Meishan Road, Hefei, Anhui 230032, China; ^2^Liuzhou Center for Disease Control and Prevention, 1 Tanzhongxi Road, Liuzhou, Guangxi Zhuang Autonomous Region 545000, China; ^3^Jingjiu District Health Centers of Fuyang City, 162 Yingshang Road, Fuyang, Anhui 341202, China

## Abstract

**Objective:**

To compare the prevalence of HIV and associated factors for participating HIV voluntary counseling and testing (VCT) among older clients of female sex workers (CFSWs) in Liuzhou City and Fuyang City in China.

**Methods:**

A cross-sectional study was conducted and the study employed 978 male CFSWs, aged 50 years and above from October 2016 to December 2017. All participants were required to complete a questionnaire and provide blood samples for HIV testing. Multivariate logistic regression analysis was used to analyze the influential factors of using VCT program and tested for HIV.

**Results:**

The HIV infection prevalence rate was 1.2% and 0.5%, while 52.3% and 54.6% participants had ever utilized VCT service and tested for HIV in Liuzhou City and Fuyang City, respectively. The older CFSWs who ever heard of VCT program were more likely to uptake VCT program in both cities (OR_Liuzhou_ = 2.224, OR_Fuyang_ = 2.421). Participants, whose marital status was married or cohabiting (OR_Liuzhou_ = 0.548, OR_Fuyang_ = 0.495), who have stigma against individuals who are living with HIV/AIDS (OR_Liuzhou_ = 0.273, OR_Fuyang_ = 0.371), whose monthly income is more than 500 yuan (OR_Liuzhou_ = 0.622, OR_Fuyang_ = 0.600), and whose age is more than 60 years old (OR_Liuzhou_ = 0.639, OR_Fuyang_ = 0.554), were less likely to visit VCT clinics. Those who are worried about HIV-infected participants were more likely to utilize VCT services in Fuyang City (AOR = 1.838, 95%CI : 1.146‐2.948).

**Conclusion:**

Combine strategy will be needed to promote the utilization of VCT service, based on the socioeconomic characteristics of older male CFSWs in different cities of China.

## 1. Introduction

It has been reported that the number of people living with HIV/AIDS has reached 780,000 at the end of 2011 in China. The people aged 50 years and above accounted for 20.6% which was more than 41 times higher compared to the data of 2000 (0.5%) [1]. The contribution of heterosexual transmission has increased from 11.3% in 2005 to 76.3% in 2011 [1, 2]. Now, it is estimated the number of female sex workers (FSWs) in mainland China is 1.8-3.8 million and clients of female sex workers (CFSWs) are 17.7-37.1 million [3]. CFSWs are thought to play a bridging role in the spread of HIV epidemic from high-risk groups to the general population [4, 5].

Due to lack of effective vaccines or cure for HIV, voluntary counseling and testing (VCT) has been regarded as an effective intervention for prevention of HIV infection. Besides, to provision of care and treatment, VCT services promote behavior changes among high-risk groups [6]. After counseling and HIV testing services provided by the consultant in a confidential environment, the inquirers are more likely to adapt necessary measures to protect themselves and their sexual partners from HIV infection according to the consultants' advices [7]. Despite the benefits of the VCT, the utilization rate was very low, only 40% of individuals living with HIV/AIDS knew their HIV sero-status worldwide, and the VCT utilization rate in general adults was only 2.5% in general adults [8], 2.3%-3.4% in rural migrants in China [9], and about 11% in adults in 45 countries of Sub-Saharan [10]. In 2003, China established 51 VCT clinics in regions with higher prevalence of HIV. In addition, the number of VCT clinic in China had increased from 2850 in 2005 to 4963 in 2007 [11].

Guangxi Zhuang Autonomous Region located in southwest China ranked second in the cumulative number of HIV/AIDS cases among 31 provinces of China in 2010. Liuzhou, as an industrial city of Guangxi Province, has been seriously affected by the HIV epidemic; according to the 2012 data, 11,323 people were infected with HIV [12]. Anhui Province locates in central-eastern China is ranked 20^th^ in the number of new HIV infection among 31 provinces of China in 2014. Fuyang City is the most serious AIDS epidemic area in Anhui Province [13], and it was famous for AIDS epidemic with blood donation in the 1990s. A number of studies have shown that the proportion of elderly HIV/AIDS cases in the total number of reported cases is increasing year by year, and the main route of transmission is heterosexual sexual contact transmission, among which commercial sexual behavior is the main one [14–16]. According to the study, the HIV infection rate of elderly clients in Guangxi from 2012 to 2013 was 2.8%, 1.94%, and 2.96%, respectively, while the proportion of elderly clients using condoms in commercial sex was less than 4% [17–19]. According to the analysis report of the AIDS epidemic in Liuzhou City from 2010 to 2015, the elderly often gather in the low-grade CSWs, the number of HIV/AIDS infection increases year by year, and the infection rate is relatively high [20, 21]. Similar to the AIDS epidemic in Liuzhou, the proportion of cases in the ≥50-year-old group in Fuyang City and Lu'an City, Anhui Province, increased significantly, reaching 32.1% and 25.0% in 2014 and 2015, respectively [22, 23]. However, the VCT outpatient utilization rate of elderly clients over 50 years old is very low [18], and whether they have been tested for HIV is a risk factor for HIV infection [19]. Even no study conducted on VCT utilization among older CFSWs in China regions, considering a high susceptibility of HIV infection and the lower utilization of VCT services among elder people, conducting a study is curtail to generate to know the prevalence of HIV and identify influential factors of utilization of VCT services among older CFSWs, and to compare between regions with higher and lower HIV prevalence.

## 2. Methods

### 2.1. Study Participants

A cross-sectional study was conducted in Liuzhou City and Fuyang City from October 1, 2016 to December 31, 2017. Health workers from local Center for Disease Control and Prevention (CDC) introduced the research team to the owners of commercial sex venues; after obtaining permission of the owner, researchers were stationed at the commercial sex venues, after brief introduction the purpose of the study to FSWs who work in the commercial sex venues and asked them to recruit eligible older male clients for this study. Participants inclusion criteria are as follows: participants who were 50 years or older, having resided in the village/community for at least 6 months, had commercial sex behavior with FSWs at least once in the past 12 months, and volunteered to participate in the study after learning the purpose of the study. Exclusion criteria are as follows: age less than 50 years old, not willing to participate in the study, not living in the city for more than 6 months, and no commercial sex behavior in the past 12 months.

### 2.2. Data Collection and Measurement

If someone was eligible, the investigator would ask for his consent to conduct an anonymous survey through face-to-face interview in a private room of the commercial sex venues. Questionnaires were checked promptly by another researcher after the interview; missing value will be input before the participants leaving the commercial sex venues. The ethics committee of Anhui Medical University approved the study protocol.

After completing the questionnaire, three milliliters of blood samples was collected by qualified nurses and transported and tested in the laboratory of local CDC within 12 hours. Plasma specimens screened for HIV antibodies by enzyme-linked immunosorbent assays (ELISA; Beijing Modern Gaoda Biotechnology Co., Beijing, China) and positive tests confirmed by a HIV-1 western blot (Diagnostics HIV Blot 2.2, Genelabs, Singapore); participants who had HIV infection were referred to their local CDC. After completing the questionnaire and blood sample collection, each CFSW was provided with 30 yuan equivalent to about U.S.$ 5 as compensation.

In this study, “Had taken VCT service” is defined as “the participants who ever taken HIV counseling and test in hospital and VCT station”. An anonymous questionnaire was used as a data collection tool. The questionnaire is designed to obtain demographic information, knowledge associated with HIV/AIDS and VCT, sexual behaviors, and attitude toward people living with HIV/AIDS. The knowledge on HIV/AIDS was measured by eight questions [24], to which participants needed to answer yes or no. Participants who responded at least six correct answers considered as had awareness on HIV/AIDS. The knowledge-related VCT measured by six questions as “Have you heard of VCT?”, “Do you know the policy of Four Free and One Care”, “Have you ever taken part in VCT program”, etc. Another six questions about attitudes to living with HIV/AIDS were used to assess stigma as “Have you worried about HIV infection?”, “Are you willing to work with HIV carriers?”, “Should AIDS patients be isolated from others?”, etc. A positive attitude for each question was scored one and a negative attitude scored zero, and the participants who got one score or more were deemed as having stigma against people living with HIV/AIDS. The questionnaire was pretested among 10 CFSWs who volunteer to take HIV test in VCT clinic of Liuzhou City; approximately 20 minutes was needed to complete each questionnaire, and these 10 CFSWs were not included in the study sample.

### 2.3. Statistical Analysis

The HIV test results and response to the questioner were entered into a database twice and matched using EpiData 3.1 (The EpiData Association, Odense, Denmark) and analyzed using SPSS 17.0 (SPSS Inc., Chicago, IL, USA). The chi-square test and Fisher's exact test were used for the proportional variable test. Measurement data of normal distribution were described with mean and standard deviation; two independent sample *t*-tests were used to compare measurement data between two cities. Multivariate logistic regression was performed to evaluate associated factors of utilization of VCT services; variables significant (*P* < 0.10) in the univariate analysis were included in a multivariate logistic regression model and *P* < 0.05 was regarded as statistically significant (two-tailed test).

## 3. Results

### 3.1. Demographic Characteristics

In total data, 575 from Liuzhou City and 403 Fuyang City eligible participants were included in this study. Most participants were less than 60 years old in both cities; the age ranged from 50 to 85 years old. More than 60% participants were married or cohabiting with a female partner.

Among Fuyang City's participants, local residents accounted almost 355 (88.10%) and one-fourth of respondents completed junior middle or lower school. While respondents from Liuzhou City, 80% of them were unemployed and more than half of them had monthly income 500 yuan (76.9 U.S.$). The majority of participants were Han ethnicity, but there were significant differences on ethnic origin between Liuzhou City and Fuyang City (Table 1).

### 3.2. Knowledge Related to HIV, VCT Service, and Stigma

Among the participants of the study, over 80 percent of participants were knowledgeable on HIV in both cities, whereas only half of the participants are worried about those who are infected with HIV. Misconceptions were common for incorrect HIV transmission routes, as demonstrated by almost 40 percent of participants who are not sure HIV cannot be transmitted by mosquito bites in two cities. Despite the high portion of participants who were aware about HIV, only 301 (52.3%) and 220 (54.6%) participants had ever utilized the VCT service and tested for HIV in Liuzhou City and Fuyang City, respectively. The VCT utilization rate was higher among participants who are aware of HIV; there were 246 (53.5%) and 184 (56.4%) participants who were aware of HIV that ever utilized the VCT service in Liuzhou and Fuyang City, but there was no significant difference between those who answered correctly less than 6 questions in Liuzhou (*P* = 0.278) and Fuyang City (*P* = 0.125).

Among the participants of Liuzhou City and Fuyang City, only 308 (53.6%) and 233 (57.8%) had heard of VCT service and received VCT service 301 (52.3%) and 220 (54.6%), respectively, but there was no significant difference on the rate of VCT utilization between the two cities (*P* = 0.489). Among those who had heard of VCT service, over 65% of them had received VCT service; participants of Liuzhou City had significantly higher rate of belief that the personal information in VCT clinics was confidential and knew where to get VCT service than that of participants in Fuyang City. Of the individuals from Liuzhou City and Fuyang City, 43.8% (252) and 21.6% (87) got a zero score for the six questions about stigma; participants of Liuzhou City (56.2%) had significantly lower rate of stigma (total score ≥ 1) than Fuyang City (78.4%) (*χ*^2^ = 51.737, *P* < 0.001) (Table 2).

### 3.3. The Prevalence of HIV and Sexual Behavior

Among participants of study, HIV test positive in Liuzhou City was 7 (1.2%) and 2 (0.5%) in Fuyang City. Though the HIV positivity rate was higher in Liuzhou City, there is no significant difference compared with Fuyang City (*χ*^2^ = 0.676, *P* = 0.411).  Table 3 shows that the prevalence of HIV infection was lower among the participants with awareness of HIV-related knowledge, and there were significant differences in both cities (Liuzhou *P* = 0.003 and Fuyang *P* = 0.036). Moreover, the participants who ever received VCT service had slightly lower rate of HIV infection in both cities, though there were no statistical significant differences (Liuzhou *P* = 0.375 and Fuyang *P* = 0.206).

The participants reported that over 53% of them had sexual intercourse with regular partners in both cities in the last six months. During sex intercourse with regular partners, the rate of consistently using condoms is only 9.3% and 10.7% in Liuzhou City and Fuyang City, respectively. The rate of commercial sex intercourse with FSWs in the last six months was 48.9% in Liuzhou City and 44.9% in Fuyang City, whereas only 13.2% of them in Liuzhou City and 15.5% in Fuyang City have always used condoms. The proportions of participants using alcohol, aphrodisiac, and illegal drugs before and during sex with FSWs in Liuzhou City were 10.6%, 8.0%, and 2.6%, while those in Fuyang City were 11.4%, 5.2%, and 0.5%, respectively.

### 3.4. Factors Associated with the Utilization of VCT

The univariate analysis showed that the utilization of VCT has strongly associated with those who are worried about HIV infection, marital status, stigma against the individual living with HIV/AIDS, monthly income, age, residential status, and ever heard of VCT service in both cities; besides these, ethnic origin was related with ever taken of VCT services in Liuzhou City, while the utilization of VCT was associated with occupation in Fuyang City (Table 4).

Multivariate logistic regression analysis showed that factors associated with the utilization of VCT service among older CFSWs were different between Liuzhou City and Fuyang City. In both cities, older CFSWs who ever heard about VCT service were more likely to receive the service in Liuzhou (AOR = 2.015, 95%CI : 1.368‐2.968) and Fuyang (AOR = 2.299, 95%CI : 1.453‐3.638). Participants who are married or cohabiting, have stigma against individuals who are living with HIV/AIDS, whose monthly income is more than 500 yuan, and whose age is more than 60 years old were less likely to visit VCT clinics. Those who worried about HIV infection participants were more likely to utilize VCT services in Fuyang City (AOR = 1.838, 95%CI : 1.146‐2.948) (Table 5).

## 4. Discussion

The aim of this study was to compare factors associated with the utilization of VCT service and prevalence of HIV among older CFSWs in two cities with higher and lower HIV prevalence. In the present study, the prevalence of HIV infection among older CFSWs of Liuzhou City was over twice higher than that of Fuyang City, which was in line with the HIV prevalence of FSW of Guangxi Zhuang Autonomous Region and Anhui Province in national HIV sentinel surveillance report [25]. The HIV prevalence of both cities was lower than that in Sichuan Province [26] and Yunnan Province [27] of China; the discrepancy may be due to the different sampling methods to recruit the samples. Almost half of the participants had sexual behaviors with regular partners and commercial sex workers in both cities, but only few of them always use condoms during sex intercourse; it indicates that there is potential risk of HIV transmission from FSW to regular partners through CFSW.

The study found the participants who had awareness of HIV-related knowledge and ever took VCT service with a lower HIV prevalence in both cities. The awareness rate of HIV-related knowledge was high (≥80%) in both cities, but the rate of VCT utilization in both cities was not in accordance with the rate of HIV-related knowledge awareness; findings of the study show that more than 65% participant who ever heard of VCT service had took part in VCT program, but only less than 60 percent of participants had heard of VCT service, even though, quite a few people do not believed the personal information was confidential and do not know where to get VCT service, which reflect that the HIV/AIDS education program among CFSW should change the direction; CFSWs know relatively well about HIV transmission methods, but they need more education about HIV test and VCT program.

The older male CFSWs with regular sexual partners were less likely to take VCT services because they were afraid that their regular sexual partners would know that they had commercial sex. The participants who have stigma against people living with HIV/AIDS were less likely to use VCT services, which was an agreement with the findings from other studies. Stigma always hinders the access to HIV testing [9, 28, 29], thus failing to disclose their health conditions. Therefore, educational messages should be more focused on people who have regular sexual partners and intend to reducing stigma against people living with HIV/AIDS, which will reduce fear of stigma among older male clients and promote the willingness to take VCT services. The participants whose monthly income was more than 500 yuan were less likely to use VCT services, which was similar with a previous study on the clients aged 50 years and over of female sex workers in Guangxi Province [30]. This may be due to the fact that the clients with higher income had higher prevalence of stigma against people living with HIV/AIDS (66.1% *vs.* 44.2%, *χ*^2^ = 27.093, *P* < 0.001). Those participants who worried about HIV infection were more likely to utilize VCT services in Fuyang City. This suggests that worrying about HIV infection is a promoting factor for elderly clients to use VCT services, and it is important to improve the risk awareness education among elderly clients to promote their active VCT testing. The data form Fuyang City showed lower rate of using VCT service among older people. More than half of participants had heard of VCT service in both cities and over two-thirds of them utilized VCT service. This indicates that the education on mode of transmission of HIV and education on VCT service were an effective intervention to improve VCT service utilization and HIV testing for the CFSWs. Moreover, it was not sufficient for promoting only uptake VCT service and attention should be given for reducing stigma against peoples with HIV/AIDS, suggesting that more efforts needed to promote VCT knowledge and HIV testing among older CFSWs.

In summary, there is potential risk of HIV transmission from FSW to regular partners through CFSW. Our findings provide important implications for the prevention of the HIV epidemic among older male CFSWs in China. At first place, some new and more receptive intervention methods should be taken according to the population characteristics of older male CFSWs in different cities. Secondly, many older male CFSWs were also having sex intercourse with their regular sexual partners; interventional methods for HIV should be expanded to cover their regular sexual partners. Thirdly, health practitioners of VCT clinics should use all strategies to improve older male CFSWs' HIV-related knowledge and eliminate stigma against people living with HIV/AIDS, thus making older male clients easily access VCT services.

The limitations of this study were the cross-sectional study method limited our ability to investigate the factors associated with having ever been tested for HIV and willingness to uptake VCT services. Secondly, convenience sampling and snowball strategies were used to recruit participants in our study, which was difficult to generalize the findings to other male client populations in China. Thirdly, commercial sex behavior is illegal in China; the sensitive questions in the questionnaire may have led to misreporting of personal attitudes and behaviors due to social desirability bias.

## Figures and Tables

**Table 1:** The sociodemographic characteristics of participants^a^.

Variable	Liuzhou City (*n* = 575)	Fuyang City (*n* = 403)	*χ* ^2^/*t*	*P*
Age (years)	59.227.66	59.067.62	0.301	0.763
Age group (years)			0.000	0.985
50-60	377 (65.6)	264 (65.5)		
>60	198 (34.4)	139 (34.5)		
Marital status				
Single/divorced/widowed	228 (37.9)	161 (40.0)	0.095	0.758
Married/cohabiting	357 (62.1)	242 (60.0)		
Residential status				
Local residents	513 (89.2)	355 (88.1)	0.302	0.583
Others	62 (10.8)	48 (11.9)		
Ethnic origin				
Han	437 (76.0)	368 (91.3)	38.169	<0.001
Others	138 (24.0)	35 (8.7)		
Educational level				
Senior middle school or higher	146 (25.4)	95 (23.6)	0.422	0.516
Junior middle school or lower	429 (74.6)	308 (76.4)		
Occupation				
Farmer	93 (16.2)	79 (19.6)	1.922	0.166
Unemployed and others	482 (83.8)	324 (80.4)		
Monthly income(yuan)				
<500	233 (40.5)	182 (45.2)	2.088	0.148
≥500	342 (59.5)	221 (54.8)		

^a^Values are given as number (percentage) unless indicated otherwise.

**Table 2:** Awareness rate of HIV, VCT service, and stigma among participants in both cities^a^.

Variable	Liuzhou City (*n* = 575)	Fuyang City (*n* = 403)	*χ* ^2^	*P*
Awareness of HIV knowledge			0.036	0.848
Yes	80.0 (460/575)	80.9 (326/403)		
No	20.0 (115/575)	19.1 (79/403)		
Ever heard of VCT service			1.732	0.188
Yes	53.6 (308/575)	57.8 (233/403)		
No	46.4 (267/575)	42.2 (170/403)		
Ever took part in VCT program			0.479	0.489
Yes	52.3 (301/575)	54.6 (220/403)		
No	47.7 (274/575)	45.4 (183/403)		
Took VCT program among participants who heard of VCT			0.041	0.840
Yes	65.3 (201/308)	66.1 (154/233)		
No	34.7 (107/308)	33.9 (79/233)		
Believed that the personal information was confidential among participants who heard of VCT			23.316	<0.001
Yes	69.8 (215/308)	54.8 (115/233)		
No	30.2 (93/308)	45.2 (118/233)		
Known where to get VCT service among participants who heard of VCT			20.141	<0.001
Yes	70.8 (218/308)	51.9 (121/233)		
No	29.2 (90/308)	48.1 (112/233)		
Score ≥ 1 for the six questions about stigma			51.737	<0.001
Yes	56.2 (323/575)	78.4 (316/403)		
No	43.8 (252/575)	21.6 (87/403)		

^a^Values are given as percentage (numerator/denominator) unless indicated otherwise.

**Table 3:** Prevalence of HIV infection associated with HIV-related knowledge and VCT utilized in Liuzhou City and Fuyang City^a^.

Variable	Liuzhou City (*n* = 575)		*χ* ^2^	*P*	Fuyang City (*n* = 403)		*χ* ^2^	*P*
	HIV positive	HIV negative			HIV positive	HIV negative		
Awareness of HIV-related knowledge			8.686	0.003			—	0.036^b^
Yes	2 (0.4)	458 (99.6)			0 (0)	326 (100.0)		
No	5 (4.3)	110 (95.7)			2 (3.9)	75 (97.4)		
Ever took part in VCT program			0.786	0.375			—	0.206^b^
Yes	2 (0.7)	299 (99.3)			0 (0)	220 (100.0)		
No	5 (1.8)	269 (98.2)			2 (1.1)	181 (98.9)		

^a^Values are given as number (percentage) unless indicated otherwise. ^b^Analysis using Fisher's exact test.

**Table 4:** Univariate analysis on factors associated with utilization of VCT services among older CFSWs in Liuzhou City and Fuyang City.

Variable	Ever took of VCT services (Liuzhou City)				Ever took of VCT services (Fuyang City)			
	No (*n* = 274)	Yes (*n* = 301)	OR (95% CI)	*P* value	No (*n* = 183)	Yes (*n* = 220)	OR (95% CI)	*P* value
Worried about HIV infection								
No	152 (55.5)	122 (44.5)			87 (57.2)	65 (42.8)		
Yes	122 (40.5)	179 (59.5)	1.828 (1.313, 2.546)	<0.001	96 (38.2)	155 (61.8)	2.161 (1.434, 3.256)	<0.001
Marital status								
Married/cohabiting	197 (55.2)	160 (44.8)	0.444 (0.313, 0.628)	<0.001	131 (54.1)	111 (45.9)	0.404 (0.267, 0.613)	<0.001
Single/divorced/widowed	77 (35.3)	141 (64.7)			52 (32.3)	109 (67.7)		
Stigma against individuals living with HIV/AIDS								
No	60 (24.4)	186 (75.6)			46 (25.7)	133 (74.3)		
Yes	214 (65.0)	115 (35.0)	0.551 (0.486, 0.625)	<0.001	137 (61.2)	87 (38.8)	0.220 (0.143, 0.338)	<0.001
Monthly income (yuan)								
<500	78 (33.5)	155 (66.5)			56 (30.8)	126 (69.2)		
≥500	196 (57.3)	146 (42.7)	0.375 (0.265, 0.530)	<0.001	127 (57.5)	94 (42.5)	0.329 (0.218, 0.497)	<0.001
Age (years)								
50-60	146 (38.7)	231 (61.3)			93 (35.2)	171 (64.8)		
>60	128 (64.6)	70 (35.4)	0.346 (0.242, 0.494)	<0.001	90 (64.7)	49 (35.3)	0.296 (0.193, 0.455)	<0.001
Occupation								
Famers	51 (54.8)	42 (45.2)			49 (62.0)	30 (38.0)		
Unemployed and others	223 (46.3)	259 (53.7)	1.410 (0.903, 2.203)	0.130	134 (41.4)	190 (58.6)	0.432 (0.261, 0.716)	0.001
Residential status								
Local residents	237 (46.2)	276 (53.8)			154 (43.4)	201 (56.6)		
Others	37 (59.7)	25 (40.3)	0.580 (0.339, 0.992)	0.045	29 (60.4)	19 (39.6)	0.502 (0.271, 0.929)	0.026
Ethnic origin								
Han	189 (43.2)	248 (56.8)			166 (45.1)	202 (54.9)		
Others	85 (61.6)	53 (38.4)	0.475 (0.321, 0.703)	<0.001	17 (48.6)	18 (51.4)	0.870 (0.435, 1.742)	0.694
Ever heard of VCT program								
No	167 (62.5)	100 (37.5)			106 (61.2)	66 (38.3)		
Yes	107 (34.7)	201 (65.3)	3.137 (2.230, 4.413)	<0.001	79 (33.9)	154 (66.1)	3.072 (2.037, 4.632)	<0.001

**Table 5:** Multivariate logistic regression on factors associated with utilization of VCT services among older CFSWs in Liuzhou City and Fuyang City.

Variable	Liuzhou City		Fuyang City	
	AOR (95% CI)	*P* value	AOR (95% CI)	*P* value
Worried about HIV infection				
No	1.000		1.000	
Yes	1.423 (0.965,2.096)	0.075	1.838 (1.146,2.948)	0.012
Marital status				
Married/cohabiting	0.548 (0.366-0.821)	0.004	0.495 (0.307-0.797)	0.004
Single/divorced/widowed	1.000		1.000	
Stigma against individuals living with HIV/AIDS				
No	1.000		1.000	
Yes	0.273 (0.183-0.408)	<0.001	0.371 (0.230-0.599)	<0.001
Monthly income(yuan)				
<500	1.000		1.000	
≥500	0.622 (0.415-0.933)	0.022	0.600 (0.5372-0.967)	0.036
Age (years)				
50-60	1.000		1.000	
>60	0.639 (0.422-0.969)	0.035	0.554 (0.332-0.926)	0.024
Occupation				
Famers			1.000	
Unemployed and others			0.956 (0.517, 1.770)	0.887
Residential status				
Local residents	1.000		1.000	
Others	1.057 (0.573,1.948)	0.860	0.762 (0.380, 1.526)	0.443
Ethnic origin				
Han	1.000			
Others	0.681 (0.434, 1.069)	0.095		
Ever heard of VCT program				
No	1.000		1.000	
Yes	2.015 (1.368-2.968)	<0.001	2.299 (1.453-3.638)	<0.001

AOR: adjusted odds ratios; 95% CI: 95% confidence interval.

## Data Availability

The data is available upon request.
